# Vancomycin Rx stewardship training for pharmacist

**DOI:** 10.1080/20523211.2025.2525354

**Published:** 2025-07-10

**Authors:** Rahela Ambaras Khan, Tan Sin Yee, Hannah Abdul Halim, Yew Jie Min, Mohammad Ammar Hakim Osman, Chew Chia Zin, Sasheela Sri La Sri Ponnampalavanar

**Affiliations:** a Department of Pharmacy, Hospital Kuala Lumpur, Kuala Lumpur, Malaysia; b Department of Medicine, University of Malaya, Kuala Lumpur, Malaysia

## Background

### Overview of vancomycin

Vancomycin, a glycopeptide antibiotic discovered over six decades ago, remains a cornerstone for the treatment of serious Gram-positive bacterial infections particularly methicillin-resistant Staphylococcus aureus (MRSA) (Cong et al., 2020; Zeng et al., 2016). MRSA infections contribute to hospital-acquired infections and have been reported at very high rates especially in Asian countries (Kang & Song, 2013). In low- and middle-income countries (LMICs), vancomycin continues to serve as a critical first line therapy due to its cost-effectiveness, accessibility and wide range of clinical application (Cong et al., 2020). Despite the availability of novel treatment options, limitations in availability and affordability of alternative treatment reinforce vancomycin central role in antimicrobial therapy in resource-limited settings. Nonetheless, vancomycin’s narrow therapeutic window and complex pharmacokinetics (PK) demand advanced clinical expertise to ensure optimal efficacy while mitigating the risks of toxicity and antimicrobial resistance.

The stewardship of vancomycin use has become increasingly essential especially in the context of global efforts to combat antimicrobial resistance (AMR). Suboptimal dosing whether under or over exposure not only jeopardise clinical outcomes but also contributes to the selection of resistant pathogens (Pereira et al., 2022). Recognising this, the Infectious Disease Society of America (IDSA) guidelines advocate for area under curve (AUC) based on therapeutic drug monitoring (TDM) over traditional trough monitoring to better correlate exposure with clinical efficacy and nephrotoxicity risk (Rybak et al., 2020). This shift necessitates that pharmacist to have a robust understanding on principal of vancomycin pharmacokinetic/pharmacodynamic (PKPD) to ensure the effectiveness of therapy.

### Therapeutic drug monitoring (TDM) for vancomycin

TDM is essential for achieving the target AUC, ensuring therapeutic efficacy and minimising vancomycin-related toxicity (Al-Maqbali et al., 2022). Vancomycin has a narrow therapeutic index, whereby underdosing may lead to treatment failure while overdosing increases the risk of toxicity, particularly acute kidney injury and ototoxicity (Martin et al., 2010). Additionally, the duration of vancomycin therapy varies depending on site of infection and clinical indication. While some infections may require only short-term therapy, others such as deep-seated infections like prosthetic joint infections and infective endocarditis may necessitate prolonged treatment durations (Le Vavasseur & Zeller, 2022). Therefore, close monitoring and TDM are crucial to ensure optimal dosing and enhancing patient safety during vancomycin therapy.

Although vancomycin dosing guidelines are adhered, considerable interpatient variability continues to pose a challenge in attaining therapeutic vancomycin concentrations (Abulfathi et al., 2018; Gu et al., 2022). Differences in PK profile that are affected by renal function, age, body weight and clinical conditions may significantly impact vancomycin dosing (Medellín-Garibay et al., 2016). For instance, elderly patients with impaired renal function are at a higher risk of toxicity compared to younger adult patients (Barber et al., 2016). On the other hand, optimising dosing in critically ill patients is particularly challenging due to pharmacokinetic/pharmacodynamic (PK/PD) alterations that significantly affect serum drug levels. Fluid resuscitation and augmented renal clearance heighten the risk of underdosing and treatment failure, whereas poor renal perfusion in septic shock can lead to drug accumulation and toxicity. The challenges become even more complex when patients require renal replacement therapy (Abdul-Aziz et al., 2020; Roberts, 2011). Hence, a structured training module is urgently needed to enhance pharmacists’ competence in the clinical application of vancomycin, ensuring optimal outcome while minimising toxicity risks.

### Vancomycin Rx stewardship

Vancomycin Rx Stewardship is a training programme designed to equip pharmacists with the essential knowledge and competencies in applying PKPD principles in real-world clinical scenarios involving vancomycin. This six-month part-time intensive programme includes pre-training preparation, comprehensive lectures and Vancomycin Optimisation Initiative Round (VOIR) which offer core principles and practical experience in optimising vancomycin across diverse patient populations. Upon completion, pharmacists will develop expertise in optimising vancomycin dosing, route and duration of therapy, thereby contributing to improved patient outcomes.

Ultimately, strengthening pharmacist capacity in vancomycin stewardship not only improves individual patient outcomes but also supports broader institutional goals of optimising antibiotic use, reducing resistance and ensuring sustainable access to essential medicines. In LMIC settings, where laboratory and diagnostic infrastructure may be limited, the clinical skills of pharmacists in guiding vancomycin therapy are particularly crucial. This programme aims to bridge knowledge gaps, promote interdisciplinary collaboration and reinforce the pharmacist’s role as a leader in antimicrobial stewardship.

## Objectives

The Vancomycin Rx Stewardship Training Programme is designed to strengthen the integration of clinical knowledge, microbiology, pharmacology, pharmacokinetics and pharmacodynamics through comprehensive training that supports informed decision-making in management of infection. It also focuses on building antimicrobial stewardship skills by equipping pharmacists to optimise vancomycin use in terms of selection, dosing, route and duration of therapy. Practical training which includes case-based simulations and real-world applications, enhances pharmacists’ ability in managing complex clinical cases.

In addition, the programme promotes cost-effective vancomycin use to reduce healthcare expenses without compromising patient safety. By addressing the risks of vancomycin misuse, it helps to reduce antimicrobial resistance and adverse events ultimately improving patient safety outcomes.

## Criteria for trainees

Eligible trainees are registered pharmacists with an interest in clinical pharmacy. The inclusion criteria required confidence in interdisciplinary interaction, including efficient communication with physicians, nurses and other health professionals. Trainees should be willing to lead training sessions and promote continuous professional development (CPD) for other pharmacists as needed. Trainees must also be able to understand and apply clinical evidence, as well as demonstrating critical appraisal skills. These criteria ensure that trainees have the necessary knowledge and skills to participate effectively in the Vancomycin Rx Stewardship Training Programme.

## Training structure

The part-time training programme begins with a two weeks of pre-training preparation and a one-week pre-assessment phase. The core training consists of six weeks of team-based learning, which includes case-based simulations and the Vancomycin Optimisation Initiative Round (VOIR). Following this, trainees complete a one-week post-assessment phase and a 12-week hands-on experience. The programme concludes with a four-week logbook submission period. The details of each phase are outlined below:

1. **Pre-Training Preparation (2 Weeks)**
  Trainees will receive a standard set of learning slides covering technical and clinical knowledge with practical applications. The technical component will include guidance on vancomycin dosing, dilution and administration, management of infusion-related issues, strategies to optimise treatment outcomes, sampling protocols with recommended monitoring frequencies and therapeutic target ranges. The clinical and practical application component will focus on vancomycin pharmacokinetics and pharmacodynamics across general and special populations including geriatric, pediatric, critically ill, oncology, augmented renal clearance, peritoneal dialysis and orthopedic patients. This component also will also address pharmacotherapy rationalisation strategies and their implementation in clinical practice.
2. **Pre-Assessment Phase (1 Week)**
  A closed-book pre-test consisting of 50 multiple-choice questions (MCQs) will be administered over a one-and-a-half-hour period to assess trainees’ baseline knowledge. Following the pre-test, trainees will complete a learning perception survey to evaluate their attitudes and perceptions toward team-based learning within the context of vancomycin stewardship.
3. **Case-Based Simulations and Vancomycin Optimisation Initiative Round (VOIR) (6 Weeks)**
  Trainees will be divided into two groups and engaged in case-based simulations covering 12 key topics related to vancomycin use in special populations and specific indications as outlined in Table 1. These topics include augmented renal clearance, critically ill, oncology, burn, orthopedic, geriatric, chronic kidney disease, pediatric, hemodialysis, continuous ambulatory peritoneal dialysis (CAPD), ambulatory peritoneal dialysis (APD) and sustained low-efficiency dialysis (SLED) population.

**Table 1.** Case-based simulation for vancomycin Rx stewardship training.

Week	Group	Cases	Approximate time
1	A	Augmented renal clearance	3 hours
		Oncology	
		Critically ill	
		Burn	
2	B	Augmented renal clearance	3 hours
		Oncology	
		Critically ill	
		Burn	
3	A	Orthopaedic	3 hours
		Geriatric	
		Chronic Kidney disease	
		Paediatric	
4	B	Orthopaedic	3 hours
		Geriatric	
		Chronic Kidney disease	
		Paediatric	
5	A	Hemodialysis	3 hours
		CAPD	
		APD	
		SLED	
6	B	Hemodialysis	3 hours
		CAPD	
		APD	
		SLED
  Each simulation session will last approximately three hours and trainees will be required to bring a scientific calculator for pharmacokinetics calculations and vancomycin dose optimisation exercises. In addition, each trainee must participate in at least one VOIR session to observe and actively engage in clinical discussions and decision-making. Active participation in all case-based simulations and VOIR sessions is mandatory.
  Trainees are expected to identify pharmacokinetic and pharmacodynamic changes, address technical components, calculate pharmacokinetic profiles and provide rational vancomycin pharmacotherapy recommendations. The identification of vancomycin-related problems, interpretation and recommendations must align with current clinical guidelines and evidence-based practices.
4. **Post-Assessment Phase (1 Week)**
  A post-test, consisting of the questions similar to those pre-tests will be conducted to assess knowledge gained throughout the training programme. A minimum passing score of 80% is required. Trainees who do not achieve this score will be given the opportunity to retake the test after two weeks. Following the post test, trainees will also be required to complete a post-training perception survey to evaluate their perceptions and attitudes toward the training.
5. **Hands-On Experience (3 Months)**
  Each trainee will be required to manage 30 vancomycin cases in collaboration with Therapeutic Drug Monitoring (TDM) pharmacists with a focus on dosing, monitoring and patient-specific adjustments. In addition, trainees will also need to conduct one self-led VOIR session per discipline, applying their knowledge in real-world scenarios. The cases will include those with persistent subtherapeutic or toxic levels, clinical failure, patients undergoing multimodal dialysis and other complicated or challenging cases. Both the case management and VOIR sessions must be completed within three months following the post-test.
6. **Logbook Submission & Certification**
  Trainees are required to complete and submit a logbook documenting all activities, learning experiences and case management. The logbook must be submitted within one month after the training. A certificate of completion will be awarded upon fulfilling all training components which include submitting a complete logbook, achieving a minimum passing score of 80% on both the post-test and logbook assessment, successfully managing 30 vancomycin cases and conducting at least one VOIR session per discipline.

## Evaluation metrics

Evaluating the performance and effectiveness of the training programme is essential to ensure learning objectives are achieved and that trainees acquire the necessary knowledge and skills. One of the most effective methods for assessing learning outcomes is through knowledge assessments, which compare pre-test and post-test results to measure knowledge acquisitions and competency improvements (Centers For Disease Control and Prevention, 2019; LeBlanc et al., 2017). This quantitative approach enables trainers and organisations to evaluate the impact of their training initiatives and to identify specific areas that requiring further development or reinforcement.

In addition, monitoring clinical outcomes and adverse drug events (ADEs) is critical in evaluating the effectiveness of training programme, particularly those related to vancomycin use. Competency assessment in vancomycin management is essential to ensure safe and effective patient care. Trainees are expected to demonstrate the ability to initiate, monitor and adjust vancomycin therapy based on individual patient needs. This involves interpreting clinical data, identifying appropriate indications for therapy, and making informed treatment decisions.

A sound understanding and application of pharmacokinetic principles are fundamental for optimising vancomycin dosing. Trainees should be proficient in calculating key parameters such as volume of distribution (Vd), elimination rate constant (Ke) and AUC. Additionally, they should be capable of interpreting TDM results, understanding the relationship between AUC/MIC ratios and therapeutic efficacy and making appropriate dose adjustment.

Based on clinical assessments, trainees should be able to provide evidence-based recommendations for vancomycin therapy including dosing modifications, monitoring strategies and collaborative interventions with the healthcare team to optimise patient’s outcomes.

Finally, evaluating changes in trainees’ perceptions and attitudes towards team-based learning, alongside collecting detailed feedback is essential for a comprehensive assessment of the training programme’s effectiveness. This dual approach offers valuable insights into both the collaborative learning process and the overall training experience.

## Conclusion

In conclusion, effective vancomycin stewardship is essential to ensure safe, optimal and patient-centered antibiotic therapy. Through this Vancomycin Rx Stewardship training programme, pharmacists are expected to apply their knowledge of pharmacokinetics and pharmacodynamics principles in managing vancomycin therapy and tailoring treatment plans based on individual patient factors. Ultimately, strengthening vancomycin stewardship practices not only enhances patient outcomes and minimises adverse effects but also supports hospital efforts in combating antimicrobial resistance and promoting overall healthcare sustainability.
